# Association between Anemia and Stroke in Females: A Nationwide, Population-Based Cohort Study in Taiwan

**DOI:** 10.3390/ijerph17207440

**Published:** 2020-10-13

**Authors:** Yuan Sui, Chien-Tai Hong, Li-Nien Chien, Hung-Yi Liu, Hung-Yi Chiou, Yi-Chen Hsieh

**Affiliations:** 1 Department of Neurology, Shuang Ho Hospital, Taipei Medical University, Taipei 11031, Taiwan; b101100004@tmu.edu.tw (Y.S.); ct.hong@tmu.edu.tw (C.-T.H.); 2 Department of Neurology, School of Medicine, College of Medicine, Taipei Medical University, Taipei 11031, Taiwan; 3 School of Health Care Administration, College of Management, Taipei Medical University, Taipei 11031, Taiwan; lnchien@tmu.edu.tw (L.-N.C.); dieseabook@gmail.com (H.-Y.L.); 4 Health and Clinical Research Data Center, Office of Data, Taipei Medical University, Taipei 11031, Taiwan; 5 School of Public Health, College of Public Health, Taipei Medical University, Taipei 11031, Taiwan; hychiou@tmu.edu.tw; 6 Master Program in Applied Molecular Epidemiology, College of Public Health, Taipei Medical University, Taipei 11031, Taiwan; 7 PhD Program of Neural Regenerative Medicine, College of Medical Science and Technology, Taipei Medical University, Taipei 11031, Taiwan; 8 PhD Program in Biotechnology Research and Development, College of Pharmacy, Taipei Medical University, Taipei 11031, Taiwan

**Keywords:** stroke, anemia, population-based cohort study, competing risk analysis, adjusted sub-distribution hazard ratio

## Abstract

Optimal stroke prevention strategies for women should take into account specific sex-related stroke risk factors. Anemia is a common medical condition in females, particularly in women of reproductive age. This study investigated whether anemia is an independent risk factor for stroke in females in a population-based cohort study. We investigated newly diagnosed anemic female patients with no history of central nervous system disease, psychiatric disorders, traumatic brain injury, major operations or hemorrhagic diseases identified from the Taiwan National Health Insurance Research Database. Non-anemic matched controls (1:1) were selected based on a propensity score estimated using a logistic regression model that included demographic characteristics and comorbidities. A competing risk analysis was applied to estimate the stroke risk in anemic patients compared to that of their matched controls. In our study, the adjusted sub-distribution hazard ratios (aSHRs) of overall, hemorrhagic and ischemic stroke in anemic female patients aged <50 years were 1.35 (95% confidence interval (CI): 1.19–1.52, *p* < 0.001), 1.31 (95% CI, 1.09-1.56, *p* < 0.003), and 1.35 (95% CI, 1.15–1.58, *p* < 0.001), respectively, compared to non-anemic female controls. However, a positive association between anemia and stroke was not found for those aged ≥50 years. Similar results were observed when the follow-up age was limited to 50 years to reduce the potential effects of menopause on stroke. In conclusion, the present population-based cohort study found that anemia is a potential risk factor for overall, hemorrhagic and ischemic stroke in females of reproductive age.

## 1. Introduction

Cerebrovascular disease, a devastating disease worldwide, ranks as the fourth most common cause of death and presents a significant disease burden in Taiwan [1]. The early screening and control of traditional vascular risk factors including hypertension, diabetes mellitus, smoking and hyperlipidemia are essential for both primary and secondary prevention [2,3], especially in young and middle-aged populations who have a longer life expectancy. Interestingly, these risk factors are less commonly found in fertile-aged female patients [4]. Specifically, differences in endogenous hormones, exogenous estrogens and pregnancy exposure are risk factors exclusively experienced by women [5], and previous research has suggested that estradiol protects the brain from experimental stroke to a great extent [6,7,8]. Nevertheless, unexpected results were encountered when translating these favorable data from the bench into clinical trials [9,10].

Anemia is a prevalent medical disorder, especially for women. The common symptoms of anemia include paleness, fainting and fatigue. Acute anemia usually occurs in patients with a large amount of blood loss, but chronic anemia may have different etiologies, including malnutrition, chronic illness and repeated occult blood loss. Recently, anemia was proven to be an independent risk factor for several systemic diseases, including cardiovascular diseases, cerebrovascular accidents and neurodegenerative diseases [11,12,13,14,15,16]. Regarding reproductive-aged women, who are less likely to have stroke due to the possible protection provided by estrogen, iron-deficiency anemia (IDA) has been previously reported to be associated with stroke [17,18,19]; however, there is a lack of strong evidence regarding the association between these two conditions obtained from large-scale cohorts or population-based study.

Although females have been shown to have a lower risk of stroke, especially at reproductive age, there are still some mysteries and unexplained cryptogenic strokes which have occurred. The identification of other unknown risk factors specifically for females is essential for better stroke prevention. Based on the previous clinical findings about stroke in patients with anemia, we aim to delineate the association between stroke and anemia—a common, easily detectable and potentially treatable prevalent medical condition—in women. 

## 2. Materials and Methods

### 2.1. Data Source

The National Health Insurance (NHI) Research Database (NHIRD) is a claims database derived from the NHI program, which was launched by a compulsory legislative act in 1995. The database contains comprehensive information on medical services that cover over 99% of the population living in Taiwan. Data collected in 2000–2016 were analyzed in this study. Disease diagnoses in the NHIRD adopted the International Classification of Diseases, Ninth Revision, Clinical Modification (ICD-9-CM) before 2015 and use the Tenth Version (ICD-10-CM) from 2016 on. In addition, the content of the database includes treatment procedures, prescribed medications, patient demographic information, reimbursement amounts and prescribed medications, which can be classified based on the Anatomical Therapeutic Chemical (ATC) Classification System for Medications. The NHI administration verifies the accuracy of the diagnoses and rationale for treatments by routinely sampling a portion of NHI claims. Furthermore, the NHIRD data are also linked to the National Death Registry using encrypted identifiers, meaning that we were able to obtain death records of subjects in our study under Health and Welfare Data Science Center regulations. This study was approved by the Taipei Medical University Joint Institutional Review Board (N201602041).

### 2.2. Study Cohort

Patients with at least two diagnostic claims for anemia (ICD-9-CM: 280.1, 280.8, 280.9, 281.x, 284.01, 284.09, 284.8, 284.9, and 285.9) or iron supplement medication (ATC: B03A) between 2002 and 2014 were first included in the study cohort. Of these patients, those diagnosed with anemia between 2000 and 2001 were excluded to ensure that our subjects were new incident cases. Furthermore, patients who did not receive at least one blood test service claim within 3 months before the index date of the anemia diagnostic claim were excluded to confirm that anemia was active. In Taiwan, generally speaking, a hemoglobin value of less than 12 g/dL is recognized as anemia. There was no significant change of the definitions regarding anemia and the measurement of hemoglobin in Taiwan during this period of time. The first claim date of an anemia diagnosis was defined as the index date. The remaining subjects who did not have a diagnosis claim of anemia were regarded as potential controls.

A pseudo date of an anemia diagnosis was randomly assigned to controls corresponding to the pool of index dates of anemia patients since they did not have an index date. The same exclusion criteria for both cases and controls were used: being aged less than 20 years; not being a citizen of Taiwan; being male or missing sex information; having been diagnosed with a central nervous system disease, psychiatric disorder or traumatic brain injury; having other diseases or medical conditions that were likely to cause anemia, such as gastrointestinal bleeding disorders and chronic renal disease, or having undergone major surgical procedures before the index date; having a diagnosis of uterine disorders or coagulation/hemorrhagic diseases; and having died or been diagnosed with a stroke within 6 months after the index date or pseudo date of anemia diagnosis. These criteria were applied to exclude people with either a higher risk of stroke or anemia due to an acute illness.

**Figure 1:**
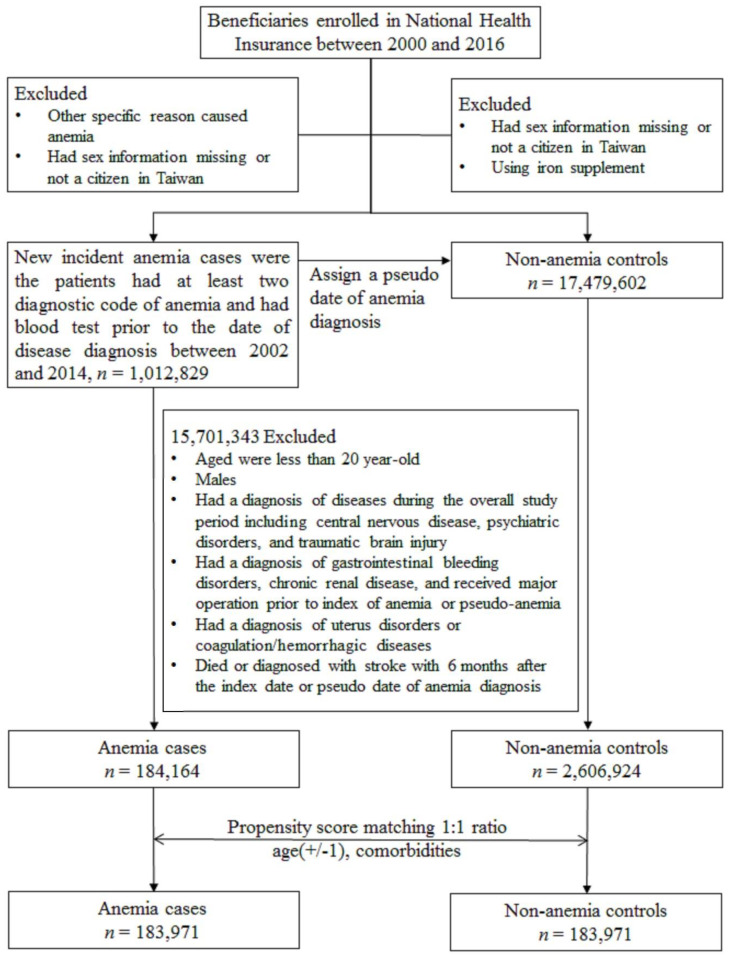
Patient selection flowchart.

Finally, propensity score matching (PSM), which is commonly used in epidemiologic studies in observational data to minimize sample selection bias, was employed. Each anemic patient was matched with an anemia-free control based on their propensity scores, which resulted in similar baseline characteristics between the two groups [20,21,22]. Propensity scores were estimated based on the variables listed in Table 1, which are factors associated with stroke according to a claim-based study. The detailed selection process is illustrated in Figure 1.

### 2.3. Main Outcome

The main outcome was a discharge diagnostic claim of stroke (ischemic stroke, ICD-9-CM: 433–434, 436; hemorrhagic stroke, ICD-9-CM: 430–432) after a neuroimaging examination. The accuracy of the stroke diagnosis using ICD coding in the NHIRD was validated in previous studies and showed high sensitivity and positive predictive values [23]. The follow-up period was defined from the anemia index date after a 6 month washout period to the date of stroke hospitalization, the date of death or 31 December 2017, for both anemic and non-anemic study subjects.

### 2.4. Statistical Analysis

Potential confounders at the baseline between anemic cases and matched non-anemic controls were compared using the standardized mean difference (SMD). A value of the SMD of ≤0.1 denoted a negligible imbalance in potential confounders between the two study groups [24]. A competing risk analysis was adopted to accurately estimate the risk of study outcomes, since death was a competing risk, especially for anemic patients. Therefore, we conducted a cumulative incidence function and sub-distribution hazard model to precisely estimate the risk of stroke. All analyses were performed using SAS/STAT 9.4 (SAS Institute, Cary, NC, USA), STATA 15 (Stata Corp LP, College Station, TX, USA), and R (vers. 3.2.5 for Windows). A two-sided *p* value of <0.05 was considered significant.

## 3. Results

The present study identified 184,164 newly diagnosed anemic female patients who fulfilled the inclusion and exclusion criteria between 2002 and 2014 from the NHIRD. After the 1:1 PSM, 183,973 anemic cases and 183,973 non-anemia controls were selected with a mean follow-up period of 9.58 ± 4.01 years. Table 1 shows that both groups had similar demographic distributions, including age and common comorbidities, after PSM. The mean age of study participants in both groups was 38.4 ± 13.4 years. The most common comorbidities were hypertension (5.5%), followed by diabetes (2.9%) and hyperlipidemia (2.4%).

**Figure 2:**
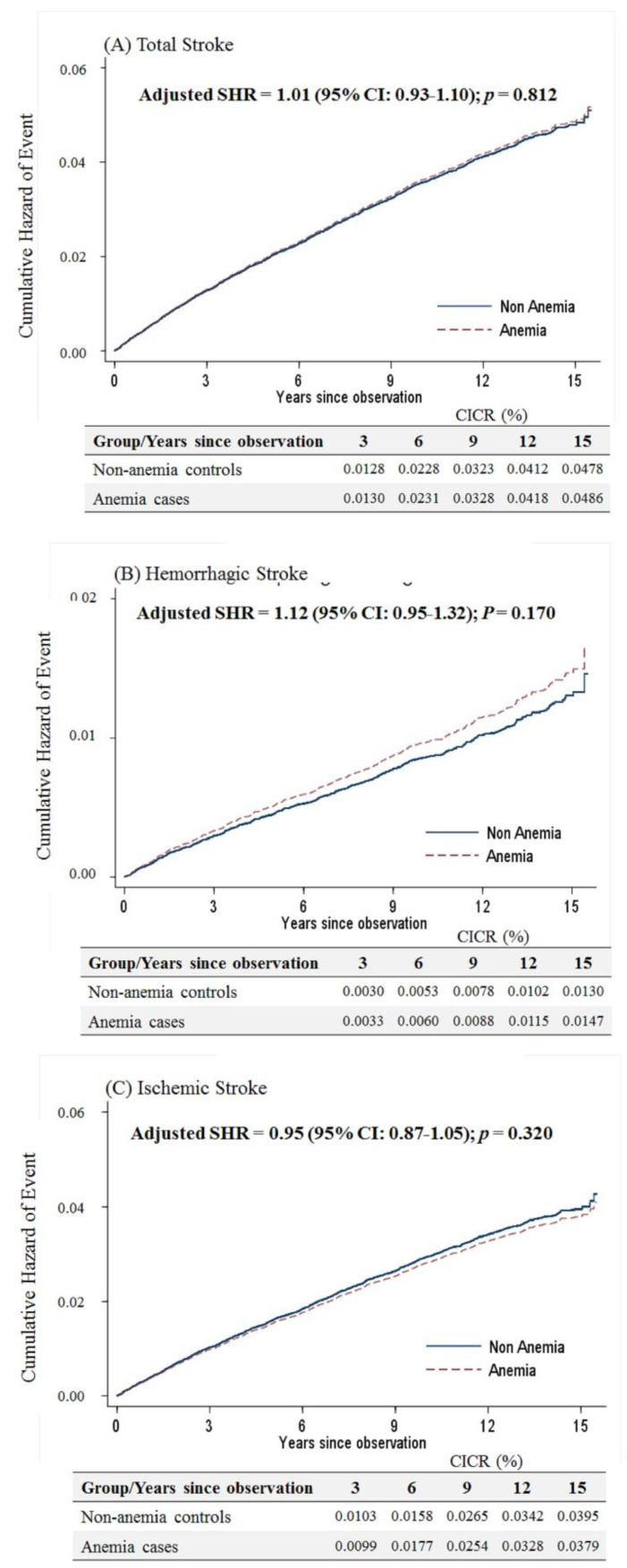
Cumulative incidences of competing risks (CICRs) of (**A**) total stroke, (**B**) hemorrhagic stroke and (**C**) ischemic stroke in anemia cases and non-anemia controls aged ≥50 years.

Considering that menopause is a risk factor for stroke in women, the present study separated the study participants into those older and younger than 50 years. For women aged ≥50 years, anemia was not associated with the occurrence of overall stroke based on the competing risk model (sub-distribution hazard ratio (SHR) = 1.01, 95% confidence interval (CI): 0.93–1.10, *p* = 0.812). Likewise, there was no difference in either the hemorrhagic or ischemic stroke risk for elderly anemic women (Figure 2).

**Figure 3:**
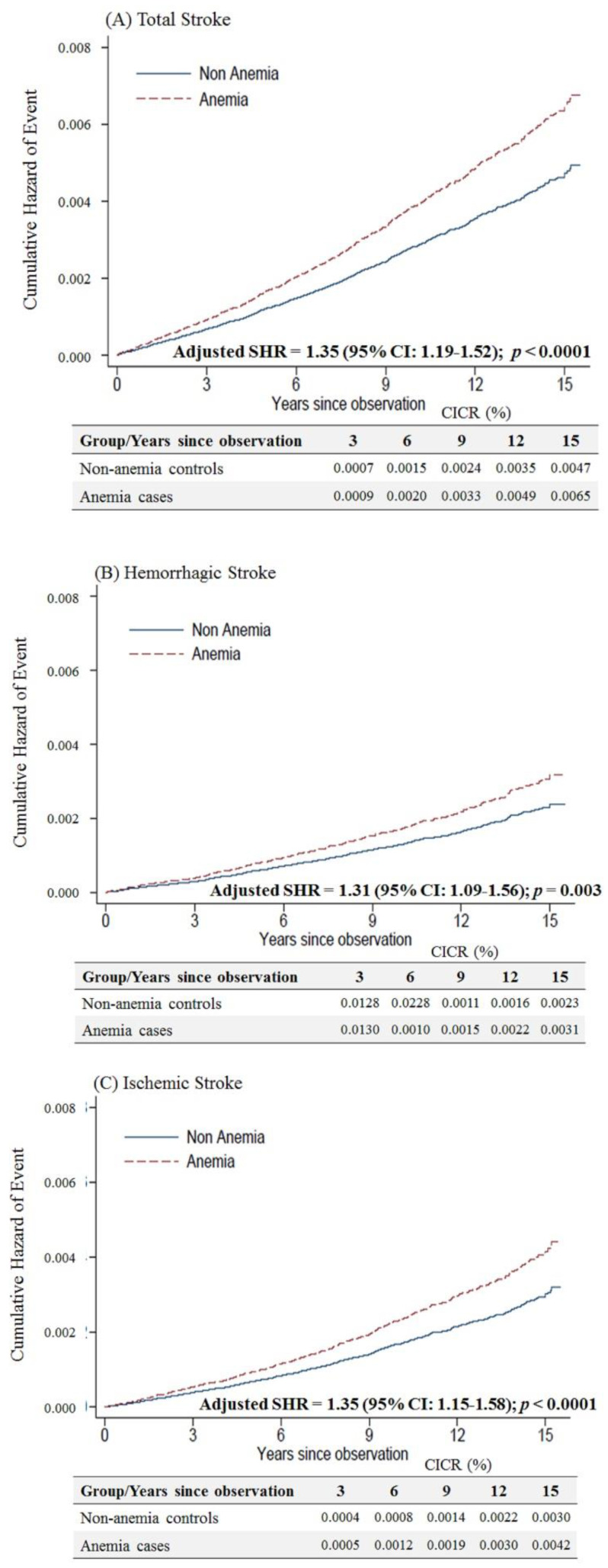
Cumulative incidences of competing risks (CICRs) of (**A**) total stroke, (**B**) hemorrhagic stroke and (**C**) ischemic stroke in anemia cases and non-anemia controls aged <50 years.

Female anemic patients aged < 50 years were at a higher risk of overall (adjusted SHR = 1.35, 95% CI: 1.19–1.52, *p* < 0.001), hemorrhagic (adjusted SHR = 1.31, 95% CI: 1.09–1.56, *p* = 0.003) and ischemic stroke (adjusted SHR of 1.35, 95% CI: 1.15–1.58, *p* < 0.001) than non-anemic controls (Figure 3).

**Figure 4:**
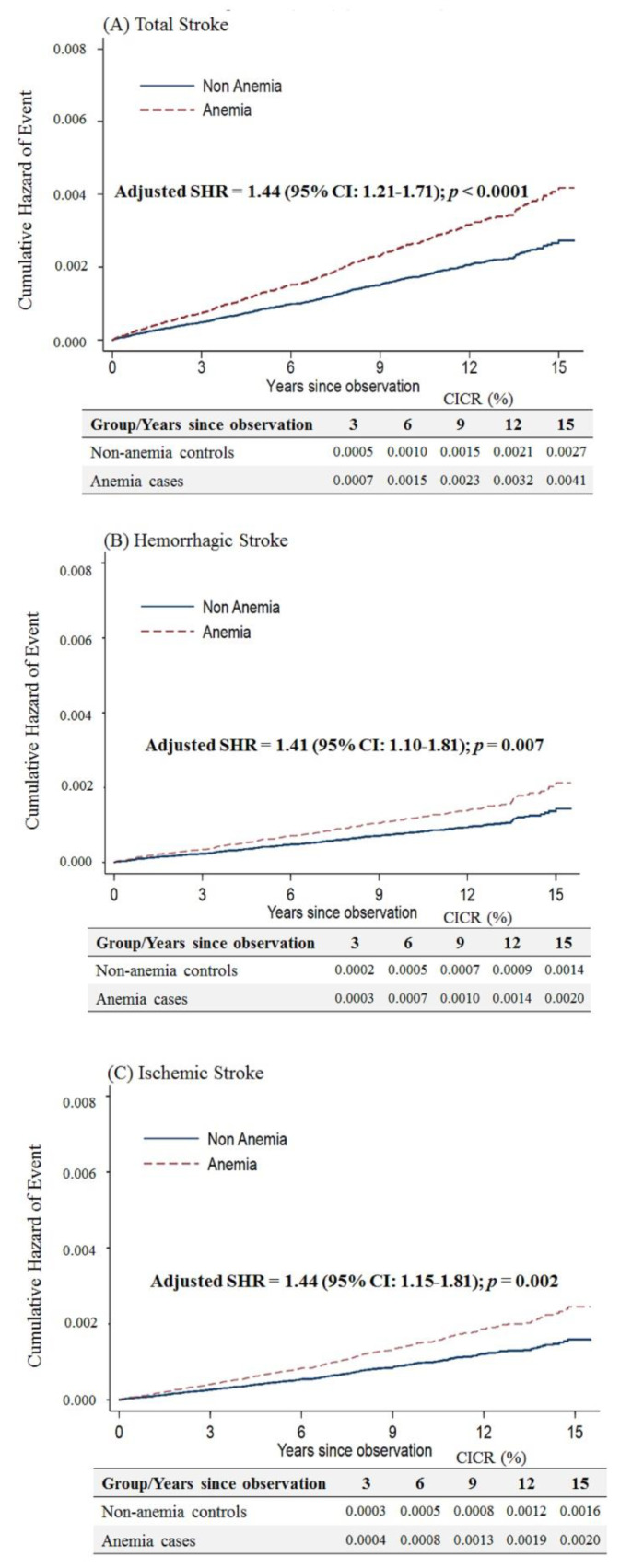
Cumulative incidences of competing risks (CICRs) of (**A**) total stroke, (**B**) hemorrhagic stroke and (**C**) ischemic stroke in anemia cases and non-anemia controls aged <50 years and limited to a follow-up period up to 50 years of age.

Considering that anemic female patients under 50 years of age would have a higher risk of stroke after menopause, we limited the follow-up period up to an age of 50 years. We still observed that anemic patients had a higher risk of overall stroke (adjusted SHR = 1.44, 95% CI: 1.21–1.71, *p* < 0.001), hemorrhagic stroke (adjusted SHR = 1.41, 95% CI: 1.10–1.81, *p* = 0.007) and ischemic stroke (adjusted SHR = 1.44, 95% CI: 1.15–1.81, *p* = 0.002) (Figure 4).

## 4. Discussion

The present study demonstrated that anemia was associated with a higher risk of stroke in reproductive-aged females; however, the association was not found in anemic female patients who were aged over 50 years. Our findings indicate that anemia should be regarded as a risk factor for stroke in women of fertile age.

Several theories have proposed that anemia affects coagulation, hemodynamics and the process of atherosclerosis [12,25,26,27,28,29]. There is some dispute as to whether hematocrit levels influence bleeding tendencies by interfering with platelet adhesion and thrombin generation. There is a positive correlation of ischemic stroke and a negative correlation of hemorrhagic stroke with hematocrit levels. However, in the Hisayama study, low hematocrit levels increased both ischemic and hemorrhagic stroke risks [30]. Thus, the theory mentioned above might not fully explain the pathophysiological U-shaped associations between these two factors. Other theories have suggested that an anemic state may result in thrombocytosis and hypercoagulability [11,26]. Iron-deficiency anemia (IDA), the primary cause of non-hemorrhagic anemia in young females—especially non-pregnant women—has been proposed to increase thromboembolic events. Some authors have concluded that transferrin overexpression due to IDA might enhance the thrombosis potential by inactivating antithrombin through the thrombin/Factor XIIa complex [31,32,33].

Both ischemic and hemorrhagic stroke partially share a similar pathophysiology with atherosclerosis of narrowing the arterial lumen and damaging arterial elasticity. From a cellular perspective, decreased hemoglobin reduces the oxygen supply to tissues and can cause hypoxia. When arteries are exposed to hypoxia, monocytes/macrophages and T lymphocytes are signaled through endothelial cells, smooth muscle cells and fibroblasts, eventually accelerating the atheroma process [34,35]. Furthermore, hypoxia impairs the mitochondrial respiratory chain, markedly disturbing energy production. Neurons are highly oxygen-demanding and may be sensitive to such hypoxic events.

Based on our study, there was a higher risk of all strokes in reproductive-aged female anemic patients. Because menopause and low estrogen levels can lead to marked physiological changes [36], we divided the participants into two age groups based on the average age at menopause (50.2 ± 4.0 years) from a large-scale health examination database in Taiwan [37]. It is well known that estrogen has a protective effect against atherosclerosis, either by ameliorating metabolic syndromes, reducing low-density lipoprotein (LDL) or homocysteine [38] and decreasing oxidative stress to vessel walls. We assumed that there was no significant difference in stroke risks between the two age groups because of more decisive factors than anemia alone. These patients were more vulnerable to ischemic and hemorrhagic stroke because of decreased estrogen and the higher prevalence of other comorbidities [36]. 

According to our results, there was an intriguing finding that anemic females of fertile age are at higher risk of developing hemorrhagic stroke. As mentioned in previous studies, common etiologies of hemorrhagic stroke in young patients are hypertension and congenital cerebrovascular malformations (arteriovenous malformations, aneurysms and cerebral cavernous venous malformations) [39]. In the present study, the impact of hypertension was adjusted for by the PSM to avoid such interference. Regarding vascular malformation-related hemorrhagic stroke, previous studies have shown that estrogen may be a protective factor against aneurysms and arteriovenous malformation ruptures, whereas pregnancy and the perineum were triggers of hemorrhagic stroke [40]. Hypocholesterolemia, an independent risk factor for hemorrhagic stroke, may be seen in chronic anemic patients with increased erythropoiesis [41]. Levels of low-density lipoprotein (LDL) and triglycerides play essential roles in the stability of cell membranes. These are inversely associated with hemorrhagic stroke by causing erythrocyte fragility, decreased platelet aggregability and arterial fragility [42]. These findings might explain the higher risk of hemorrhagic stroke in reproductive-aged anemic females.

The strength of this study is that almost all of the Taiwanese population is covered in the NHIRD. In addition, any diagnoses in the health insurance database were rigorously made under the government’s policies, ensuring our study’s validity. Second, we excluded patients who had had a stroke within 6 months after the index date of anemia to reduce the bias by the simultaneous diagnosis of the two diseases. We also excluded uncommon types of stroke, such as cerebral venous thromboses, which have a pathophysiology distinct from conventional arterial strokes, to find a more accurate association. Third, we applied the PSM approach to facilitate the selection of controls with an almost equal rate of baseline characteristics. Lastly, we used a competing risk model to examine the association between anemia and stroke, considering that anemic patients may possess a higher mortality rate [43], which may shorten the observation period and underestimate the incidence of stroke.

There are some limitations to our study. First, data from the NHIRD lack information on the severity of anemia, or how well patients were controlling their blood sugar and blood pressure. However, we viewed this problem with caution and only recruited anemic patients who had been confirmed twice in a 3 month interval by a blood test to guarantee that each case followed a chronic course and was potentially irreversible. Second, lifestyle is another major contributing factor to atherosclerosis for which we could retrieve no data, including cigarette and substance use and caffeine intake. In addition, nutrition data such as deficiencies in folate or vitamin B12 which may result in anemia and an increased risk of stroke are not included in the claim data. Third, we did not consider that patients with mild anemia could be relatively healthy and thus visit the clinic less frequently and thus possibly not obtain a diagnosis. Lastly, IDA cannot be correctly defined in our study, especially in women of fertile age, as there is no menstrual data for individuals in our data [44].

In summary, this population-based cohort study showed that reproductive-aged female anemia patients had an associated higher risk of stroke. Considering the socioeconomic burden of stroke, anemic females of fertile age should be informed about the greater risk of stroke and be alert to any symptoms of stroke in order to avoid delayed treatment.

## Figures and Tables

**Table 1:** Baseline characteristics of anemia cases and non-anemia controls before and after propensity score matching (PSM).

	Before Matching					After Matching				
	Control *n*	(%)	Case *n*	(%)	SMD	Control *n*	(%)	Case *n*	(%)	SMD
Sample size	2,606,924	100%	184,164	100.0%		183,971	100%	183,971	100.0%	
Age, mean (SD)	38.9 (13.4)		38.5 (13.5)			38.4 (13.4)		38.4 (13.4)		
20–39	1,444,920	55.4%	111,237	60.4%	0.101	109,562	59.6%	111,195	60.4%	0.018
40–49	595,065	22.8%	43,179	23.4%	0.015	43,292	23.5%	43,163	23.5%	0.002
50–59	366,931	14.1%	15,789	8.6%	0.174	16,884	9.2%	15,769	8.6%	0.021
60–69	140,054	5.4%	7046	3.8%	0.074	7147	3.9%	7028	3.8%	0.003
70+	59,954	2.3%	6913	3.8%	0.085	7086	3.9%	6816	3.7%	0.008
Comorbidity, yes										
Hypertension	143,174	5.5%	10,288	5.6%	0.004	10,172	5.5%	10,169	5.5%	<0.0001
Diabetes	56,471	2.2%	5511	3.0%	0.052	5400	2.9%	5407	2.9%	<0.0001
Hyperlipidemia	61,961	2.4%	4499	2.4%	0.004	4446	2.4%	4448	2.4%	<0.0001
Coronary artery disease	20,318	0.8%	2001	1.1%	0.032	1919	1.0%	1936	1.1%	0.001
Heart failure	3136	0.1%	593	0.3%	0.043	491	0.3%	533	0.3%	0.004
Atrial fibrillation	1441	0.1%	215	0.1%	0.021	149	0.1%	194	0.1%	0.008
Peripheral artery disease	1747	0.1%	208	0.1%	0.015	174	0.1%	186	0.1%	0.002
Malignant neoplasm	11,071	0.4%	2867	1.6%	0.114	2795	1.5%	2766	1.5%	0.001
Rheumatic disease	40,173	1.5%	3893	2.1%	0.043	3827	2.1%	3829	2.1%	<0.0001

Abbreviation: PSM: propensity score matching, SD: standard deviation, SMD: standardized mean difference; SMD: difference in means or proportions divided by standard error; an imbalance was defined as an absolute value >0.1.

## Abbreviations

ASHR	Adjusted sub-distribution hazard ratio
ATC	Anatomical Therapeutic Chemical
CI	Confidence interval
ICD-9-CM	International Classification of Diseases, Ninth Revision, Clinical Modification
IDA	Iron-deficiency anemia
LDL	Low-density lipoprotein
NHI	National Health Insurance
NHIRD	National Health Insurance Research Database
PSM	Propensity score matching
SMD	sSandardized mean difference
